# Supplementary material for: Escherichia coli Isolated from Diabetic Foot Osteomyelitis: Clonal Diversity, Resistance Profile, Virulence Potential, and Genome Adaptation
Source: Microorganisms. 2021 Feb 13;9(2):380. doi: 10.3390/microorganisms9020380 (PMC7918245; doi:10.3390/microorganisms9020380)
Supplement: Supplementary file 1 [file microorganisms-09-00380-s001.pdf]

Supplementary information

Table S1. Ecology of Diabetic Foot Infections during the inclusion period of the study in Nîmes University Hospital

Microorganism(s)	N (%)	Multidrug-resistant bacteria* (%)
**Aerobic Gram-positive cocci**	327 (46.5)	
Staphylococcus aureus	162 (23.0)	28 (17.3)
Enterococcus faecalis	27 (3.8)	–
Other *Enterococcus*	3 (0.4)	–
Coagulase-negative Staphylococcus	57 (8.1)	–
Streptococcus spp.	78 (11.1)	–
**Aerobic Gram-negative bacilli**	203 (28.8)	
Enterobacteria	181 (25.7)	15 (8.3)
Escherichia coli	36 (5.1)	3 (8.3)
Proteus mirabilis	33 (4.7)	3 (9.1)
Enterobacter cloacae	30 (4.3)	4 (13.3)
Morganella morganii	27 (3.8)	2 (7.4)
Klebsiella oxytoca	14 (2.0)	1 (7.1)
Citrobacter spp.	13 (1.9)	1 (7.7)
Serratia spp.	11 (1.6)	1 (9.1)
Proteus spp.	7 (1.0)	–
Others	10 (3.2)	–
Stenotrophomonas maltophila	5 (0.7)	5 (100)
Pseudomonas spp.	7 (1.0)	3 (42.9)
Others	10 (3.2)	1 (10)
**Gram positive aerobic bacilli**	37 (5.3)	
Corynebacterium striatum	24 (3.4)	
**Anaerobes**	134 (19.0)	
**Fungi**	3 (0.4)	

*Multidrug resistant bacteria included methicillin-resistant *S. aureus*, *Enterobacteriaceae* resistant to third-generation cephalosporins, *P. aeruginosa* or *Acinetobacter baumannii* resistant to ceftazidime and *Stenotrophomonas maltophilia*.

Table S2. *Escherichia coli* toxin/antitoxin and virulence factors encoding ORF

Strain	Toxin/Antitoxin ORF	Other virulence ORF
NECS21	Toxin HokB (x2)<br>Antitoxin Ccda<br>Antitoxin HigA-2 (x3)<br>Cytoskeleton-binding toxin CbtA (x2)<br>Persistence and stress-resistance antitoxin PasI<br>Ribosome association toxin RatA<br>Toxin GhoT (x2)<br>Antitoxin PrlF<br>Toxin YhaV<br>Toxin-antitoxin biofilm protein TabA (x3)<br>Antitoxin ChpS<br>Antitoxin RelB	Glutamate decarboxylase (x2)<br>Salmonella HilA homolog<br>Enteroaggregative immunoglobuline repeat protein<br>Long polar fimbriae
NECS50	Toxin HokB (x2)<br>Antitoxin Ccda<br>Antitoxin HigA-2 (x3)<br>Cytoskeleton-binding toxin CbtA (x2)<br>Persistence and stress-resistance antitoxin PasI<br>Ribosome association toxin RatA<br>Toxin GhoT (x2)<br>Antitoxin PrlF<br>Toxin YhaV<br>Toxin-antitoxin biofilm protein TabA (x3)<br>Antitoxin ChpS<br>Antitoxin RelB	Glutamate decarboxylase (x2)<br>Salmonella HilA homolog<br>Enteroaggregative immunoglobuline repeat protein<br>Long polar fimbriae
NECR70	Toxin HokB (x2)<br>Antitoxin HigA-2 (x2)<br>Cytoskeleton-binding toxin CbtA (x2)<br>Persistence and stress-resistance antitoxin PasI	Glutamate decarboxylase<br>Salmonella HilA homolog<br>Enteroaggregative immunoglobuline repeat protein

	Ribosome association toxin RatA<br>Toxin GhoT (x2)<br>Antitoxin PrlF<br>Toxin YhaV<br>Toxin-antitoxin biofilm protein TabA (x2)<br>Antitoxin MqSA	Long polar fimbriae
NECR107	Toxin HokB (x2)<br>Antitoxin HigA-2 (x2)<br>Cytoskeleton-binding toxin CbtA (x2)<br>Persistence and stress-resistance antitoxin PasI<br>Ribosome association toxin RatA<br>Toxin GhoT (x2)<br>Antitoxin PrlF<br>Toxin YhaV<br>Toxin-antitoxin biofilm protein TabA (x2)<br>Antitoxin MqSA	Glutamate decarboxylase (x2)<br>Salmonella HilA homolog<br>Enteroaggregative immunoglobuline repeat protein<br>Long polar fimbriae

Table S3. Effect of single-nucleotide polymorphism on the *Escherichia coli* strain firstly isolated.

		High effect		Moderate effect	Low effect
**Strain**	**SNP**	**Start lost**	**Stop gained**	**Missense**	**Synonymous**
NECS21	1,764 (1,277 genes)	1 (1 gene)	29 (22 genes)	561 (127 genes)	472 (121 genes)
NECR70	6,874 (2,198 genes)	6 (6 genes)	132 (99 genes)	2,721 (373 genes)	2,155 (358 genes)

Numbers represent the count of identified single-nucleotide polymorphism
